# Remodeling adipose tissue through in silico modulation of fat storage for the prevention of type 2 diabetes

**DOI:** 10.1186/s12918-017-0438-9

**Published:** 2017-06-12

**Authors:** Thierry Chénard, Frédéric Guénard, Marie-Claude Vohl, André Carpentier, André Tchernof, Rafael J. Najmanovich

**Affiliations:** 1 Department of Pharmacology and Physiology, Faculty of Medicine, Université de Montréal, Montreal, QC Canada; 2 Department of Biochemistry, Faculty of Medicine and Health Sciences, Université de Sherbrooke, Sherbrooke, Canada; 3 Institute of Nutrition and Functional Foods, Université Laval, Quebec City, Canada; 4 School of Nutrition, Université Laval, Quebec City, Canada; 5 Division of Endocrinology, Department of Medicine, Centre de recherche du CHUS, Université de Sherbrooke, Sherbrooke, Canada; 6 Centre de Recherche de l’Institut universitaire de cardiologie et de pneumologie de Québec, Quebec City, QC Canada

**Keywords:** Metabolic network, Diabetes, Adipocytes, Lipid metabolism, Flux balance analysis, *in-silico* single gene deletion, Biomass production, Lipid droplet production

## Abstract

**Background:**

Type 2 diabetes is one of the leading non-infectious diseases worldwide and closely relates to excess adipose tissue accumulation as seen in obesity. Specifically, hypertrophic expansion of adipose tissues is related to increased cardiometabolic risk leading to type 2 diabetes. Studying mechanisms underlying adipocyte hypertrophy could lead to the identification of potential targets for the treatment of these conditions.

**Results:**

We present *i*TC1390adip, a highly curated metabolic network of the human adipocyte presenting various improvements over the previously published *i*Adipocytes1809. *i*TC1390adip contains 1390 genes, 4519 reactions and 3664 metabolites. We validated the network obtaining 92.6% accuracy by comparing experimental gene essentiality in various cell lines to our predictions of biomass production. Using flux balance analysis under various test conditions, we predict the effect of gene deletion on both lipid droplet and biomass production, resulting in the identification of 27 genes that could reduce adipocyte hypertrophy. We also used expression data from visceral and subcutaneous adipose tissues to compare the effect of single gene deletions between adipocytes from each compartment.

**Conclusions:**

We generated a highly curated metabolic network of the human adipose tissue and used it to identify potential targets for adipose tissue metabolic dysfunction leading to the development of type 2 diabetes.

**Electronic supplementary material:**

The online version of this article (doi:10.1186/s12918-017-0438-9) contains supplementary material, which is available to authorized users.

## Background

Type 2 diabetes is one of the most prevalent non-infectious diseases in the world and affects an increasing number of patients every year. The development of insulin resistance in multiple tissues and impaired insulin secretion by pancreatic β-cells in response to glucose are the two main pathophysiologic characteristics of type 2 diabetes [1]. Both are necessary conditions for the development of type 2 diabetes since a normal response of pancreatic β-cells usually compensates for insulin resistance and maintains normal blood glucose level [2]. Experimental studies in animal models and in humans show that overexposure of lean-tissues to fatty acids plays an important role in the development of insulin resistance and impaired insulin secretion by a process called lipotoxicity [3].

Exposure of lean-tissues to fatty acids is regulated by adipose tissues. Deficits in adipose tissue expansion during chronic positive caloric imbalance is linked to increased levels of circulating fatty acids that contribute to ectopic fat deposition and lipotoxicity in lean tissues [4]. Adipose tissues respond to excess energy with two types of expansion: 1) hyperplasia, which is the recruitment and differentiation of new pre-adipocytes to mature adipocytes resulting in more abundant but smaller adipocytes; 2) hypertrophy, an increase in adipocyte size through storage of lipids, resulting in fewer but larger adipocytes. Body fat distribution is usually reflective of the type of expansion favored by adipose tissues and of detrimental metabolism. Visceral fat accumulation is often associated with adipocyte hypertrophy, while accumulation of subcutaneous adipose tissues is associated with hyperplasia [5, 6]. Hypertrophic expansion of adipose tissues is often linked to the development of metabolic dysfunction, which in turn contributes to the development of type 2 diabetes [7–9].

Improved knowledge of the mechanisms leading to adipose tissue hypertrophic vs. hyperplasic expansion during weight gain could lead to the identification of new targets for the prevention and treatment of type 2 diabetes. One method for studying cells at the systemic level is the use of metabolic networks. Metabolic networks serve as a representation of metabolism at the cellular scale and can be used to identify metabolites, reactions, genes and pathways linked to various diseases [10–12]. Reactions included in metabolic networks are a mix of enzymatic, transport, exchange and spontaneous reactions.

Two genome-scale metabolic networks, Recon1 [13] and the Edinburgh Human Metabolic Network [14], were developed in 2007 to help investigate human metabolism in various contexts. Recon2 [15], a community-based expansion on Recon1, was later created based on two previous networks as well as other sources of information such as HepatoNet1 [16] and an additional extensive literature search. These networks are generic representations of human metabolism and they miss some elements specific to various cell lines such as detailed lipid metabolism, which is important in the study of the development of obesity, adipocyte hypertrophy and type 2 diabetes.

Various, more specific models were developed over the years to study the effect of diabetes in various tissues [17–19]. These networks were used to study differences between subjects with and without type 2 diabetes using either uptake and secretion rates or transcriptomic data. These analyses identified various metabolites and pathways differently regulated between the two conditions which could be used as potential markers for type 2 diabetes. Despite the interesting results obtained, the above metabolic networks lacked an explicit account of lipid droplet production and degradation pathways specifically in adipocytes. As lipid droplets management is an essential aspect of adipocyte function likely associated with the development of type 2 diabetes, the latter networks may have missed important aspects of the disease.

A genome-scale metabolic network of human adipocytes called *i*Adipocytes1809 was developed by Mardinoglu et al. [20]. It is significantly larger than the previous adipocyte networks described above and included detailed reactions for fatty acid synthesis as well as lipid droplet production and degradation pathways. The authors used expression data from lean and obese subjects to identify pathways in which gene expression varied between the two conditions. More recently, the authors developed a modified version of their network and identified Branched-chain Amino Acid Transferase (*BCAT1*) as a potential target for the treatment of obesity [21]. Unfortunately, this updated version of the adipocyte network is not freely available, preventing its use.

In this work, we generated a highly curated, freely available human adipocyte metabolic network improving on the published *i*Adipocytes1809 and used the resulting metabolic network to perform a number of analyses. Among these, we performed in silico gene deletions in which we used biomass and lipid droplet production as proxies for hyperplasia and hypertrophy respectively, to identify genes that could tilt the balance away from adipocyte hypertrophy. Furthermore, we used experimental postprandial concentration curves of circulating glucose, non-esterified fatty acids (NEFA) and triacylglycerol (TAG) to simulate the adipocyte metabolic responses over time after meals. Studying variation in the production rate of metabolites as a function of time opens the possibility to use metabolic networks as a window to the internal state of adipocytes as a function of time. Considering that a computational model of adipocyte metabolism gives access to measurements of intracellular metabolites that may be difficult to perform experimentally, this approach generates hypotheses that could lead to a better understanding of adipocyte function.

## Methods

During our use of the *i*Adipocytes1809 metabolic network we found that it had some limitations. It contains genes that are not expressed in adipocytes, various erroneous gene-reaction associations, reactions that do not occur in adipocytes and lack of critical information regarding protein complexes that are essential to the determination of the effect of in silico mutations. Furthermore, *i*Adipocytes1809 also lacked a precisely defined biomass reaction, necessary for the flux balance analysis (FBA) calculations we wished to produce [22]. FBA is a constraint-based modeling method commonly used in the study of genome-scale metabolic networks. FBA uses directionality and stoichiometry of reactions in the network to calculate flow of matter through the network, enabling the prediction of growth rate or the production rate of important metabolites. FBA is often used to predict the effect of gene deletions or modifications on the network.

The following steps were undertaken in order to generate an improved version of *i*Adipocytes1809: 1) removal of genes not expressed in adipocytes; 2) removal of incorrect gene/reaction associations; 3) removal of non-metabolic reactions; 4) reduction of the number of dead-end metabolites; 5) addition of new genes, reactions and gene/reaction associations; and lastly, 6) addition of information relative to protein complexes. These steps resulted in the creation of a new network, *i*TC1390adip. The network was validated using a battery of 245 tests and used in various analyses. These steps are described next.

### Removal of genes not expressed/translated in adipocytes

We used the *i*Adipocytes1809 network supplementary data provided by Mardinoglu et al. [20] to remove as many genes not expressed in adipocytes as possible from the network. These data included human protein atlas [23] data as well as the author’s own proteomics data and information derived from literature searches. In addition to the above, we used gene expression data from Affymetrix HG-U133A DNA microarrays corresponding to 8 obese, non-diabetic, normolipidic human male adipose tissue samples (4 donors, both subcutaneous and visceral tissues) [24]. We called each gene as either present, absent or putatively present in adipocytes. In order to include as many genes as possible in our analysis, we considered a gene as being expressed if the gene was defined as being expressed or putatively expressed in at least one of the tissue samples.

Genes not tested in any of the sources of expression data described above were kept in the network. Genes for which all available data indicated that the gene was not expressed were removed from the network. If such a gene was the only one related to a particular reaction, that reaction was removed from the network, otherwise it was retained. For example, the Carboxy Ester Lipase (*CEL*) gene, which is expressed in the pancreas and mammary glands and encodes an enzyme excreted into the digestive tract and breast milk [25], was present in the *i*Adipocytes1809 network even though all expression data available indicated that this gene is not expressed in adipose tissue. Unexpressed genes whose removal would block the production of biomass or lipid droplet were kept in the network. We also removed the synthesis of certain hormones such as thyroxine and melatonin for which there is no evidence of production by adipocytes [26, 27].

### Correction of gene/reaction associations

We used the Enzyme Commission Numbers (ECN) [28] associated to the reactions performed by the enzymes encoded by the genes present in *i*Adipocytes1809. The ECNs associated to each gene in the network was obtained from NCBI using the NCBI gene identifier of each gene as well as data available on GeneCards [29] to manually verify suspicious associations. Suspicious associations occur when the gene and reaction are not associated to the same complete ECN as well as all those with missing or incomplete ECN. Associations considered as erroneous were removed from the network. For example, the R-type/L-type Pyruvate Kinase (*PKLR*) gene, responsible for phosphorylation of pyruvate as part of glycolysis (EC: 2.7.1.40), was associated with glutathione degradation converting glutathione into cysteinyl-glycine and glutamate (EC: 3.4.19.13) even if the gene and reaction are not linked in any ways. Some associations in which the gene and reaction had the same ECN were also removed when the reaction was not consistent with the specificity of the gene product. For example, the Succinate-CoA Ligase, ADP-Forming, Beta Subunit (*SUCLA2*) gene is an ATP-dependent succinate-CoA ligase which was associated to the GTP-dependent version of the reaction.

### Removal of non-metabolic functions

Several pathways and reactions in the *i*Adipocytes1809 network are not directly involved in metabolism, as for example, protein synthesis and degradation, post-transcriptional modification, DNA methylation, *PPARA* activation. Although all molecular processes are interconnected, we proceeded with the assumption that the elements above should not be part of a metabolic network because they lead to a blurring of the conceptual boundaries between intracellular sub-systems (metabolism, regulation, signaling, etc.) as well as an artificial inflation of the size of the network. For example, *i*Adipocytes1809 contains 62 different genes linked to protein-L-tyrosine phosphorylation. We manually verified pathways and metabolites to identify those linked to such non-metabolic functions and removed the appropriate genes and reactions.

#### Reducing the number of dead-end metabolites

*i*Adipocytes1809 as well as the partially curated network following the steps above contained a large number of dead-end metabolites. Dead-end metabolites are defined as metabolites that can only be either consumed or produced within the network. All input metabolites to the network need to be provided and are dead-ends by definition but are necessary. Likewise, output metabolites (i.e., biomass components) are also dead-end but their production is the objective of the simulated network. Unlike these, dead-end metabolites within the network are biological impossibilities. The detection of dead-end metabolites involves standard graph analysis techniques and are part of the different packages to analyze biological networks. We used the Sybil package version 1.2.6 [30]. Some dead-end metabolites were already in the network while others were created by removal of reactions during one of the previous curation steps. For example, the removal of protein degradation in the peroxisome turned the transport of amino-acids from the peroxisome to the cytosol into a dead-end, since there is no source of amino-acid in the peroxisome. Some dead-ends were removed while others were connected by addition of the necessary reactions (and their associated genes) or exchange reactions. The addition of such reactions may introduce genes in the network for which there is no evidence of expression. However, given the biological impossibility of dead-ends, it is more likely that expression data was lacking.

### Addition of genes and reactions from KEGG and Recon2

Using the gene expression data described above, we identified genes expressed in adipose tissue that were not present in the *i*Adipocytes1809 network. We searched KEGG [31] and Recon2 [15] for reactions associated to those genes and added to the network the genes and reactions that did not introduce new dead-ends. Some genes were added to reactions already in the network, increasing the number of gene/reaction associations.

### Addition of protein complexes

Protein-protein interactions are important in the network since they can change the essentiality of a gene based on the involvement of its protein product in a protein complex. Protein complexes may be permanent or transient, and in the case of the latter, involve constant or variable (at times mutually exclusive) sets of partners [32]. These details are unknown and we assumed that all complexes are permanent and that each subunit of a complex is necessary for activity, some complexes having multiple versions using different subunits. We used information from Uniprot [33] to identify genes in the network that are part of complexes and added them to the network. We also added the genes corresponding to other sub-units of the identified complexes that were missing in the network. For example, the serine palmitoyltransferase complex had some of its subunits in the network (SPTLC1, SPTLC2, SPTLC3) while the SPTLCA and SPTLCB subunits were added. These subunits generate various functioning complexes (SPTLC1 with either SPTLC2 or SPTLC3 and either SPTCLA or SPTLCB) with varying affinities for the different substrate species [34].

### Validation of iTC1390adip

Validation being an important part of a metabolic reconstruction, we submitted our network to the same 244 tests used to validate the *i*Adipocytes1809 network [20]. These tests are meant to ensure that the metabolic model can perform relevant metabolic functions and fail to generate elements that cannot be produced by the cell. For example, the synthesis of essential amino acids should not be possible. When tests failed, verifications were made to identify the source of the problem and solve it by adding the minimal number of reactions needed to successfully complete the test.

McQuaid et al. measured TAG and glucose uptake as well as NEFA release using multiple and simultaneous stable FA isotope tracers over 24 h in lean and obese subjects [35]. The creators of the *i*Adipocytes1809 metabolic network used these data in their network to test the capacity of their network to produce lipid droplet. As additional validation of *i*TC1390adip, we also used the data from McQuaid et al. to recreate the lipid droplet production estimation experiment. FBA is an equilibrium technique; therefore, it is not possible to use FBA to study time-dependent processes. In order to do so, the data from McQuaid et al. for each time step was used to constraint the flux through particular reactions and as such create unique equilibrium networks representing each individual time step. Specifically, for each time point over the 24 h period, we used experimental data to constrain the lower and upper bounds of each associated exchange reactions and to calculate optimal production of both lipid droplet and acetyl-CoA independently. Other exchange reactions need to be active for these simulations to work. For Acetyl-CoA production, O_2_ absorption as well as H_2_O and CO_2_ excretion are active and we also added inorganic phosphate, ammonia and hydrogen ion uptake during lipid droplet synthesis.

Lastly, we compared the effect of single gene deletions on biomass in our network using FBA to experimental results of gene deletion on the survival and proliferation of various human cancer cell lines. We compared our results to those of cancer cell lines, as large scale gene deletion data are not available for human adipocytes. Despite differences between cell types, broad agreement is expected. Blomen et al. [36] used gene-trap mutagenesis on the KBM7 and HAP1 cell lines, which are respectively diploid and haploid chronic myelogenous leukemia cancer cell lines, while Wang et al. [37] used CRISPR on four different cell lines, the KBM7 and K562 chronic myeloid leukemia cell lines and the Raji and Jiyoye Burkitt’s lymphoma cell lines. While the authors observed good overlap of equivalent predictions between both methods, differences in gene essentiality were found across cell lines. Therefore, we used a consensus prediction between the two publications. We created two lists of consensus gene essentiality predictions from the data in the two publications, one a consensus including all cell lines (comprising the data of six experiments using five cell lines) or a consensus using only the KBM7 cell line that was tested by both groups. The assignment of gene essentiality can be found in the Additional file 1. The gene essentiality data was used to define a threshold value for the decrease in biomass production upon the deletion of a gene (relative to the unaltered network) for which we obtain the highest level of agreement between essential and non-essential predicted genes and their experimental consensus assignment. In other words, if the deletion of a gene leads to a decrease in biomass production of below the defined threshold, that gene was said to be essential. The value of the threshold was chosen to maximize the agreement with the predictions for single gene deletions and the consensus experimental data discussed above. Interested readers may utilize the R code provided in Additional file 2 to run simulations and the validation tests of if iTC1390adip described above.

### Gene deletion analysis

We used our network to predict which genes have an effect on biomass and lipid droplet production using two different growth conditions. The growth media composition used for the network was based on Iscove’s Modified Dulbecco’s Medium (IMDM) [38] in which we replaced soybean lipids with triglycerides of the same composition as human very-low-density lipoprotein and chylomicrons. IMDM is commonly used for the culture of human cell lines and was used by both Blomen et al. [36] and Wang et al. [37] to test the effect of gene deletions. The complete list of metabolites used as part of our simulation of the IMDM growth medium is available in Additional file 3: Table S1. The tested conditions consisted of the IMDM growth medium with and without restriction of glucose and TAG uptake. To simulate the restriction, we restrained the upper values of the exchange reactions to the uptake values of McQuaid et al. [35] for lean subjects at the 11 h time point after meal since it was the time point with the greatest overall production of lipid droplet and acetyl-CoA. We did the simulations with and without restriction on TAG and glucose uptake to simulate various limiting elements, simulations without restriction have ATP production as the limiting factor while those with restrictions have carbon availability as the limiting factor. We tested the optimal production of both biomass and lipid production in the deletions using FBA and compared these values to the values obtained with the undeleted network. We used relative changes in biomass and lipid droplets as a proxy for hyperplasia and hypertrophy respectively.

### Definition of biomass and lipid droplets

The biomass reaction is used in metabolic network analysis to simulate cellular growth. The biomass reaction is a simplified representation of the production of the major macromolecules and metabolites necessary to produce a new cell. Specifically, biomass consists of proteins (in terms of the amino acid composition of the proteome of the given cell), nucleic acids (RNA and DNA in terms of their composition) and cellular membranes. The biomass components capture the major components of a cell but should not be considered more than a rough estimate. We used a modified version of the biomass definition from the Recon2 metabolic network [15]. The modifications that were made to the definition of the biomass were to the cellular membranes because *i*TC1390adip (as well as *i*Adipocytes1809 network) uses different definitions for lipids consisting of diacylglycerols, TAG, cholesterol and cholesterol-esters as well as a variety of phospholipids. Adipocyte biomass usually includes the lipid droplet but since our goal is to identify genes affecting lipid droplet production without affecting cellular metabolism, we decided to remove the lipid droplet from our definition of biomass. The objective function used for the production of lipid droplets the same used by the iAdipocyte1809 network [20]. In our analysis, we used biomass and lipid droplet production as proxies for hyperplasia and hypertrophy in adipocytes respectively. The biomass and lipid droplet production reactions are described in detail in Additional file 3: Table S1.

### Metabolic differences between subcutaneous and visceral adipose tissues

We used flux variance analysis (FVA) to identify the maximum fluxes attainable by each of the network’s reactions during unrestricted optimization of biomass and lipid droplet production. FVA is a variation of FBA in which the network returns a range of possible fluxes for each reaction which are compatible with the optimization of the objective function instead of a single optimized solution. FVA uses the same objective functions (biomass) as FBA. Using the same DNA microarray data we used to identify reporter metabolites and the maximal fluxes obtained using FVA, we restricted the maximal flux of each reaction using the smallest expression fold difference of genes associated to the reaction, keeping the maximal flux of the reaction in the tissue with more expression to the value obtained by FVA and dividing that value by the fold difference for the other tissue. If a reaction had genes more highly expressed in both tissues we considered the reaction as neutral and did not change its maximal flux value. Using this method, we produced two networks with maximal fluxes representing subcutaneous and visceral adipose tissues. We did the same gene deletion analysis on both of these networks then on the *i*TC1390adip network.

All validations and simulations in this article except for the reporter metabolites analysis were performed using the Sybil package version 1.2.6 [30] available for the freely available R environment for statistical computing (version 3.0.2). Other tools are available to run this kind of simulation like the COBRA [39] and RAVEN [40] package which run on the proprietary Matlab computing environment.

## Results

### Modification of the network

Comparing the list of genes in *i*Adipocytes1809 to the proteomic data provided with the network, we identified 272 genes where proteomic data indicated that the gene was not expressed in adipocytes. Independently, 388 genes were indicated as not expressed when comparing the genes in the network with the DNA microarray data. Comparing the two sources of data, we obtained 77 genes that were not expressed in any sample, 153 genes that were not expressed but only tested in one of the analyses and the remaining 353 genes considered as unexpressed in one analysis and expressed in the other. Of the 230 genes considered as unexpressed, Ornithine Carbamoyltransferase (*OTC*), Elongation of Very Long Chain Fatty Acids Protein 4 (*ELOVL4*), Mevalonate Decarboxylase (*MVD*) and Phosphoribosylformylglycinamide Synthase (*PFAS*) were kept in the network because their removal blocked the production of biomass. The genes above are essential to enable biomass and lipid droplet production but we were unable to find alternative pathways that could compensate their function.

The ECNs of each gene was compared to that of each reaction associated to any given gene. Of the 14,985 associations present, 1439 had different ECNs for the gene and reaction, 1693 had incomplete ECNs for the gene, the reaction or both, 5029 had no ECN associated with at least one of the elements and the remaining 6824 had the same ECN for both the gene and reaction. Of those 14,985 associations in the network, we removed a total of 3583 after individual verifications using KEGG, NCBI, GeneCards and Brenda. From the removed associations, 1137 had different ECNs for the gene and reaction, 1332 and 1056 respectively had incomplete or no ECN for at least one of the elements and 58 had the same ECN for both gene and reaction but with different specificity. Removal of these associations eliminated 660 reactions and 332 genes. Removal of hormone syntheses not occurring in the adipocyte led to removing 44 reactions and 3 genes. We kept the conversion of the inactive thyroxine to the active triiodothyroxine as well as the import and export of those metabolites because these activities are known to occur in adipocytes [41].

From the list of genes expressed in adipocytes identified in the DNA microarray analysis that were not present in the network, we searched in the KEGG database and the Recon2 human metabolic network for reactions associated to those genes. This search led to the addition of 93 reactions associated to 49 new genes as well to 45 genes already present in the network. Twenty-four of the new genes and an additional 105 new genes were added to the network and associated with reactions which were already present. The use of interaction data from Uniprot about all the genes in the network led to the addition of 473 complexes to the network, including 223 genes already in the network, 7 genes added previously and 62 new genes, multiple complexes being variants of the ATP synthase complex which has a lot of redundancy in subunits. There was a total of 49 unique complexes, which are involved in various enzymatic reactions.

The removal of non-metabolic elements from the network that included but was not limited to protein production, degradation and post-transcriptional modification, DNA methylation and *PPARA* activation, reduced the size of the network without affecting the results of our FBA analysis. This step included 442 metabolites taking part in 499 reactions leading to the removal of 418 genes. Additionally, to reduce the number of dead-ends, we added 8 reactions back to the network with 5 genes including 3 new genes. We also added 58 exchange reactions to the network for dead-end metabolites that were extracellular and should be able to be exchanged with the environment. During this step, we also removed an additional 197 reactions and 22 genes pertaining to dead-ends.

### Validation of iTC1390adip

**Fig. 1:**
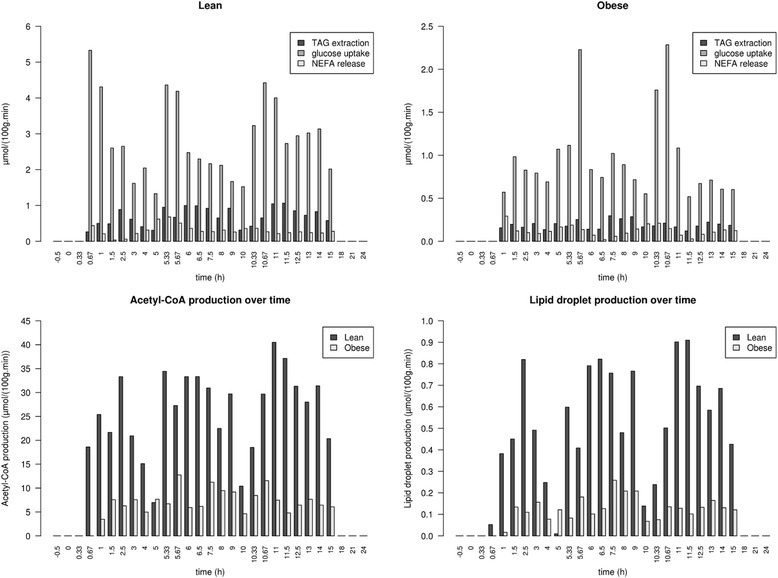
Simulation results for acetyl-CoA and lipid droplet production in obese and lean subjects using the McQuaid et al. data [35] on TAG and glucose uptake as well as non-esterified fatty-acid release as upper and lower bounds. Top-left and top-right graphs respectively represent the experimental lean and obese uptake and release rates calculated in the McQuaid et al. article used to restrict exchange boundaries. *Lower-left* and *lower-right* graphs respectively represent the predicted optimal production of acetyl-CoA and lipid droplets by lean and obese subjects during the 24 h period. *Dashed lines* represent the time at which subjects consumed controlled meals containing the stable fatty acid isotopes used to follow fatty acid absorption

Initially, 17 of the 244 tests (Additional file 3: Table S2) used to validate *i*TC1390adip failed but the reintroduction of 13 genes removed in the first step of curation led to all the tests being successful, except thioredoxin synthesis (Table 1). The initial validation steps were the same that were used for the validation of the *i*Adipocytes1809 metabolic network [20]. Running the validation on the *i*Adipocytes1809 metabolic network shows that arginine degradation fails due to the absence of urea in the simulation conditions and the absence of associated transport and exchange. The network was unable to generate thioredoxin, which was expected as we removed protein synthesis from our network. To compensate, we added to the validation a test to insure the network could generate all amino acids from the same inputs that were used to create thioredoxin. The simulations of lipid droplet and acetyl-CoA production using the uptake data from McQuaid et al. [35] generated results that were similar to those obtained using the *i*Adipocytes1809 network, where lower lipid droplet and acetyl-CoA production was observed in obese versus lean subjects during the postprandial period, except at the 5 h time point (Fig. 1 and Additional file 3: Tables S3-S4). After these modifications, the resulting network, *i*TC1390adip, contained 1390 genes coding for proteins involved in 4914 reactions using 3664 metabolites (1907 unique metabolites) including 195 exchange metabolites. The *i*TC1390adip network is available in SBML format usable with the sybilSBML package [30] for the R computing environment and in SBML usable by the RAVEN toolbox [40] for Matlab. The RAVEN compatible version is available on the BioModels website [42] with the identifier MODEL1603110000.

**Table 1:** List of tests which failed and the genes added to correct them

Failed validation^a^	Gene(s) added to correct
UDP-N-acetyl D-galactosamine de novo synthesis	GNPNAT1, GALE
CMP-N-acetylneuraminate de novo synthesis	GNPNAT1
N-Acetylglucosamine de novo synthesis	GNPNAT1, RENBP
Galactose degradation	GALE
UDP-galactose de novo synthesis	GALE
Lactosylceramide de novo synthesis	GALE
Cysteine de novo synthesis	CBS
Methionine degradation	CBS
Homocysteine degradation	CBS
CoA de novo synthesis	CBS, SLC5A6
GSH de novo synthesis	CBS
Histidine degradation	FTCD, UROC1
Phenylalanine degradation	HGD
Tyrosine degradation	HGD
Tryptophan degradation	HAAO, AFMID, ACMSD, SLC25A21
Heme de novo synthesis	SLC46A1

List of validation tests which wrongly failed in the metabolic network and that were fixed by the addition of identified genes and associated reactions

*GNPNAT1* Glucosamie-Phosphate N-Acetyltransferase 1, *GALE* UDP-Galactose-4-Epimerase, *RENBP* Renin Binding Protein, *CBS* Cystathionine-Beta-Synthase, *SLC5A6* Solute Carrier Family 5 Member 6, *FTCD* Formimidoyltransferase Cyclodeaminase, *UROC1* Urocanate Hydratase 1, *HGD* Homogentisate 1,2-Dioxygenase, *HAAO* 3-Hydroxyanthranilate 3,4-Dioxygenase, *AFMID* Arylformamidase, *ACMSD* Aminocarboxymurconate Semialdehyde Decarboxylase, *SLC25A21* Solute Carrier Family 25 Member 21, *SLC46A1* Solute Carrier Family 46 Member 1

^a^ A list of all validations is available in Additional file 3: Table S2

### Determination of the threshold at which the decrease in biomass is associated with impaired cellular function

**Fig. 2:**
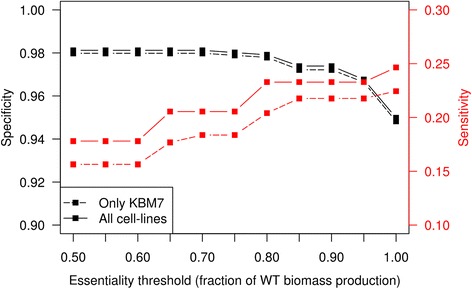
Graph representing the specificity and sensitivity of network predictions compared to experimental deletions using consensus between cell lines in two combinations of experiments. Only KBM7 uses the results of the KBM7 cell lines from both experiments. All cell lines include all six experiments from these analyses [36, 37]

We compared the effect of gene deletions in the metabolic network to the experimental effect of gene deletion in various cancerous cell lines [36, 37] in order to determine the threshold at which biomass reduction best correlates with impaired cell function. We used two different combinations of tests to compare our predictions (both KBM7 cell lines and all cell lines). For both combinations, we considered only genes for which there is consensus between all the cell lines considered. We calculated the accuracy of our prediction against the experimental data using increasing values of biomass reduction as the cutoff for predicted essentiality for optimal cell growth. We obtained the highest levels of correct predictions when using 80% of the wild type biomass production as the cutoff. We obtained a specificity of 97.8% and 97.9%, a sensitivity of 20.4% and 23.3% and an overall accuracy of 88.2% and 92.6% for KBM7 and all cell lines respectively (Fig. 2 and Table 2). Specificity is the capacity to predict non-essential genes while the sensitivity is the capacity to predict essential genes. We therefore used 80% of the wild type biomass production as the cutoff above which gene deletions are considered as not affecting cell growth during the rest of our experiments. Various reasons may explain the low level of sensitivity obtained when comparing our predictions based on biomass production to the experimental data, including difference in cell type (as none of the cancer cell lines are derived from adipocytes), the participation of experimentally important genes in pathways not linked to biomass production or the varying binding affinities of different enzymes responsible for the same reaction (see discussion for examples).

**Table 2:** Comparison of predicted and experimental genes essential for optimal growth

80% threshold	Only KBM7			All cell lines		
Predicted\Experimental	Essential	Not essential	No consensus	Essential	Not essential	No consensus
Essential	30	23	7	17	20	23
Not essential	117	1020	160	56	939	302
	0.882	correct		0.926	correct	
	0.204	sensitivity		0.233	sensitivity	
	0.978	specificity		0.979	specificity	

These results use the 80% threshold of wild type biomass production. The “No consensus” column corresponds to genes for which the experimental results did not match for all of the experimental cell lines

### Gene deletion analysis

**Fig. 3:**
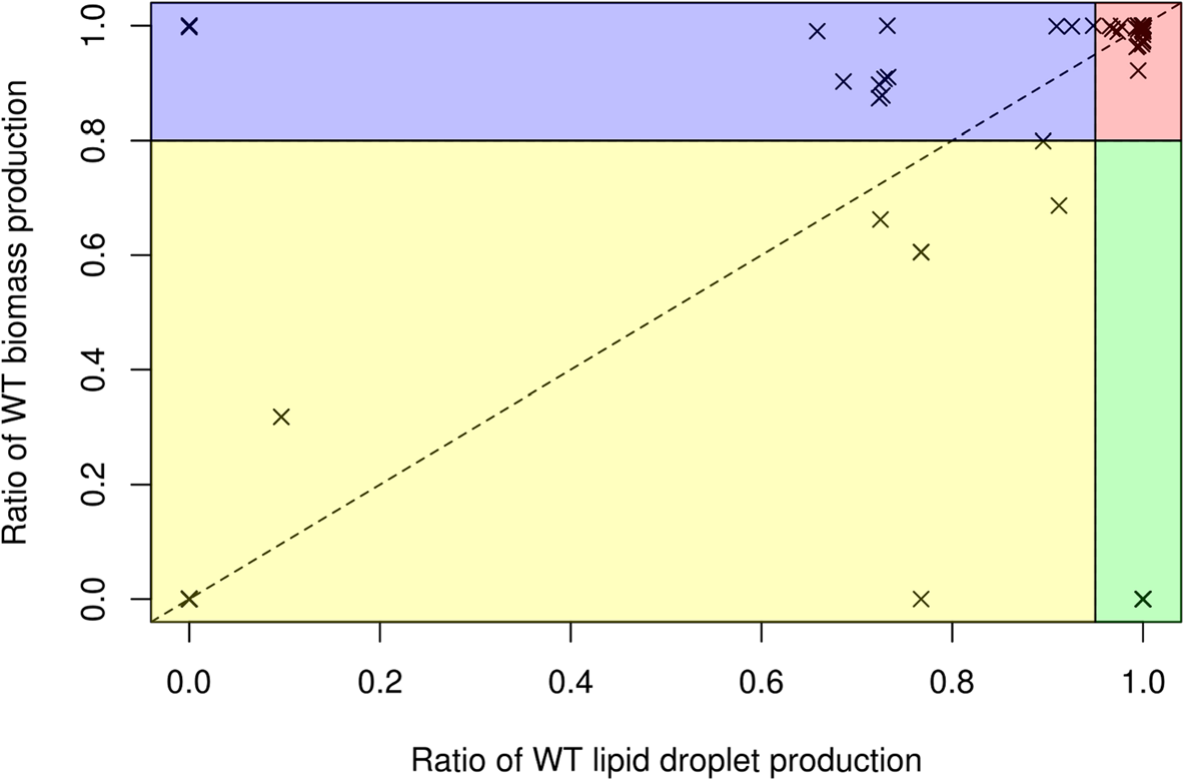
Graph of the effect of each gene’s deletion on both biomass and lipid droplet production compared to the wild type network when no restriction was applied to glucose and TAG uptake. Genes in the *blue* section are those we are interested in, they have an effect on lipid droplet production while being above the 80% threshold of biomass production. More details on these genes can be found in Table 3. The same type of graph was created for the restricted condition which is available as Additional file 3: Figure S1

With the *i*TC1390adip network in hand, we analyzed the effect of individual gene deletions on the production of both biomass and lipid droplets in order to identify genes that may negatively affect lipid droplet production with minimal impact on the production of biomass. We identified the genes associated with more than 5% reduction of lipid droplet formation while remaining above the 80% cutoff for biomass production with and without restrictions on glucose and triglyceride uptake (Fig. 3 and Additional file 3: Figure S1). A total of 27 genes were identified in either test conditions but only 3 in both. Of the remaining 24, 11 were identified only when glucose and triglyceride uptake were restricted and 13 were identified when no restrictions were applied. Despite choosing a threshold for change in biomass to minimize the detection of genes affecting cellular growth, of those 27 genes, 11 were essential for growth in at least one of the cell lines tested experimentally (Table 3): 3 in all 6 cell lines, 2 in 5 cell lines, 1 in both gene-trap mutagenesis tests and 6 in only 1 of the cell lines. For each of the 27 genes, we searched in the Online Mendelian inheritance in Man database (OMIM) [43] and in the Mouse Genome Database (MGD) [44] respectively for information on human diseases (Table 3) and mouse models associated to the gene (Additional file 3: Table S5). The absence of 18 of these genes was associated to diseases of varying severities in humans, 7 of those diseases being directly linked to lipid metabolism, and 18 have had homozygous null mouse models created. Nine of the homozygous mouse models are non-viable at birth or shortly after. Two others display significantly shorter life spans than normal mice (Additional file 3: Table S5).

**Table 3:**
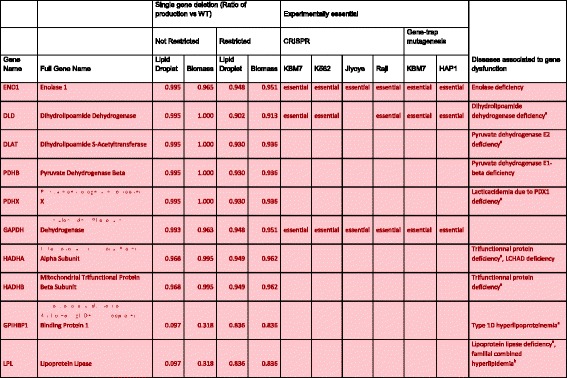
Details about the genes which could positively affect adipocyte hypertrophy

Experimental CRISPR data is from Wang et al. while the gene-trap mutagenesis data is from Blomen et al. The disease information was extracted from OMIM. Genes interesting while restricting TAG and glucose absorption are in red, those interesting while no restrictions are applied are in green and those identified in both tests are in yellow. ^A^autosomal recessive disease, ^b^autosomal dominant disease

### Metabolic differences between subcutaneous and visceral adipose tissues

Fold differences in expression between subcutaneous and visceral adipose tissues were available for 882 of the 1390 genes present in the *i*TC1390adip network. Overall, 58 genes had a stronger effect on lipid droplet and/or biomass production in one of the two tissues compared to the other (>10% difference in their effect compared to the WT) (Additional file 3: Table S6). We used the thresholds for biomass and lipid droplet production discussed above to identify genes that could have a hypertrophy-reducing effect (smaller lipid droplets) without affecting the viability of adipocytes (little or no effect on biomass production) thus shifting the fate of pre-diabetic adipocytes towards hyperplasia, a condition with reduced risk of type 2 diabetes development. Twenty different genes were identified in either tissues. Two in visceral adipose tissue, 14 in subcutaneous adipose tissue and 4 in both tissues. Of those 20 genes, only 4 were not identified in the previous analysis using the *i*TC1390adip metabolic network (1 in visceral adipose tissue and 3 in subcutaneous adipose tissue). Of those, 1 was associated to a human disease and had a mouse deletion model, 1 was only associated to a human disease, 1 was associated to a mouse deletion model and the last 1 was associated to neither. Deletion effect of all 20 genes are available in Additional file 3: Table S7, details about associated human diseases and mouse models for the 16 previously identified genes can be found in Table 3 and Additional file 3: Table S5 while they can be found in the discussion for the 4 newly identified genes.

## Discussion

The presence of inconsistencies such as genes unexpressed in adipocytes and incorrect gene-reaction associations in the *i*Adipocytes1809 metabolic network provided opportunities for extensive manual curation of the present genome-scale metabolic network reconstrution. While the automatic generation of metabolic networks [45] is an important goal, manual curation remains essential. This issue is not restricted to eukaryotic metabolic networks that are arguably more complex than prokaryotic networks [46]. Several of the interesting gene predictions with iTC1390adip could not have been made with *i*Adipocytes1809. For example, identification of Lipase A (*LIPA*) as a gene of interest would not have occurred if the *CEL* gene, which is not expressed in adipocytes, was still in the network, because they were associated to the same reactions. In some cases, we predicted complexes having an interesting effect that could not have been made with *i*Adipocytes1809 due to the absence of complexes.

When using the experimental data on gene deletion and comparing them to our prediction on the production of biomass, we obtained a maximum of 56.6% sensitivity. This low sensitivity may in part be explained by the fact that some experimentally essential genes are linked to functions not directly related to biomass production. For example, multiple subunits of the vacuolar proton pump, which is responsible for the acidification of the peroxisome and other intracellular compartments, are experimentally essential but have no effect in the network since proton accumulation leading to protein degradation is not part of the network. Another source of error is the varying enzyme kinetics for genes with the same functions. For example, the ribonucleotide reductase complex is formed of two subunits, Ribonucleotide Reductase M1 (*RRM1*) and either Ribonucleotide Reductase M2 (*RRM2*) or Ribonucleotide Reductase M2 B (*RRM2B*). Both *RRM1* and *RRM2* are experimentally essential. In our network only *RRM1* is predicted to be essential due to the presence of *RRM2B,* which can compensate *RRM2* in its absence. However, *RRM2B* cannot compensate *RRM2* experimentally due to lower catalytic activity of the *RRM1/RRM2B* complex (compared to *RRM1/RRM2)*, that is not sufficient to maintain growth experimentally [47]. Whereas such differences in rate of catalysis are fundamental experimentally, in FBA they are ignored as the role of FBA is to find the range of rates for all reactions that maximizes the production of biomass.

The use of FBA to study biomass production in human metabolic networks is not common because the optimization of biomass production is more likely to represent cancerous rather than normal human cells. We still decided to use biomass since we worked in a qualitative manner more than in a quantitative manner to gauge the differences between wild type and mutant biomass versus lipid droplet production. Our analysis is, to our knowledge the first-time multiple different objective functions were used to study the effect of gene deletions in a metabolic network study. We used biomass and lipid droplet production as representations of hyperplasia and hypertrophy, respectively. This approach allowed us to identify genes that we believe could reduce hypertrophy without affecting normal cell survival and have the potential to represent interesting targets in the modulation of fat storage and the prevention or treatment of type 2 diabetes.

Some of the potential target genes can serve as a validation of the network due to previously known implication in the development of type 2 diabetes or its complications. Mice deficient in either Diacylglycerol O-Acyltransferase 1 (*DGAT1*) or Lecithin-Cholesterol Acyltransferase (*LCAT*) are resistant to diet induced obesity and insulin resistance [48, 49] and these genes were not essential in any of the tested cell lines which makes them good potential pharmacological targets even if they are both associated to diseases in human (Congenital diarrheal disorder [50] and fish-eye disease [51] respectively). Acylglycerol kinase (*AGK*) whose disruption in humans is associated to the development of the Sengers Syndrome, an autosomal recessive disease related to abnormality of the mitochondria and in the storage of lipids and glycogen, cataracts and lactic acidosis [52], is also overexpressed in cases of diabetic retinopathies [53]. Mice with diet-induced obesity show an increased expression of Choline/Ethanolamine Phosphotransferase 1 (*CEPT1*), the muscle specific knockout of *CEPT1* led to improved insulin sensitivity and levels of *CEPT1* expression were inversely correlated with insulin sensitivity in obese human subjects [54]. 3-Hydroxy-3-Methylglutaryl-CoA Synthase 1 (*HMGCS1*) and Fatty Acid Desaturase (*FADS1*) where both identified as being overexpressed in obesity and linked to higher risks of developing type 2 diabetes [55]. *HMGCS1*, the gene coding for the protein needed for the step just before the reaction linked to 3-Hydroxy-3-Methylglutaryl-CoA Reductase (*HMGCR*), the target of statins in the cholesterol synthesis pathway, is essential for optimal growth in all the experimentally tested cell lines used during the validation of the network. *HMGCR* was also essential in all tested cell lines but still represented a good target in the treatment of metabolic abnormalities. This indicates that genes that are essential in the various cell lines should still be investigated despite the essentiality data. *FADS1* deficiency in mice leads to the synthesis of arachidonic acid-derived eicosanoids, which results in failure to thrive and death by 12 weeks of age [56]. The rest of the potential targets are not known to be associated to the development of type 2 diabetes.

Fatty Acyl CoA Reductase 2 (*FAR2*) is not associated with any disease or mouse models and was also not essential for the optimal growth of the various tested human cell lines. *FAR2* has a paralog, Fatty Acyl CoA Reductase 1 (*FAR1*), which is not expressed in adipocytes based on our gene expression data. Theoretically, targeting only *FAR2* could reduce the risk of side effects since *FAR1* would compensate the loss of function of *FAR2* in tissues other than adipocytes. *CEPT1* was essential for growth in only one of the cell lines, 6 other genes were also essential in one or two of the tested cell lines (Phosphatidylserine Synthase 1 (*PTDSS1*), Elongation of Very Long Chain Fatty Acids Protein 1 (*ELOVL1*), Trans-2,3-Enoyl-CoA reductase (*TECR*), Phosphate Cytidylyltransferase 2 (*PCYT2*), Succinyl-CoA Ligase subunit A (*SUCLG1*) and Alkylglycerone Phosphate Synthase (*AGPS*)). These 7 genes might be essential in their respective cell lines due to other mutations present specifically in these cancer cell lines. Homozygous mouse models for two of the previously mentioned genes (*PCYT2* and *ELOVL1*) die at the embryonic level or shortly after birth [57, 58]. It is unknown if the inhibition of the respective enzymes in adults would also be lethal. Four other potential targets (Enolase 1 (*ENO1*), Dihydrolipoamide Dehydrogenase (*DLD*), Glyceraldehyde-3-Phosphate Dehydrogenase (*GAPDH*) and Hydroxysteroid 17-Beta Dehydrogenase 12 (*HSD17B1*2)) are also essential in most of those cell lines and homozygous null mouse models for each of these genes die at the embryonic state [59–62]. It has been shown that HSD17B12 knock down is linked to reduced lipid accumulation in adipocytes which would match the effect predicted by the network [63]. Four additional genes (Lipoprotein Lipase (*LPL*), *ELOVL4* and the Mitochondrial Trifunctional Protein Alpha and Beta Subunits (*HADHA/HADHB*)) caused mice to die sooner than expected when they were individually deleted [64–67]. The pyruvate dehydrogenase complex is a target that could not have been identified in the original network due to the absence of information on protein complexes. However, the absence of any of the subunits (*DLD*, Dihydrolipoamide S-Acetyltransferase (*DLAT*), Pyruvate Dehydrogenase Beta (*PDHB*), Pyruvate dehydrogenase Component X (*PDHX*)) results in lactic acidemia [68–70] and *DLD* deficiency is more severe and causes other complications because it also participates in the branched-chain alpha-keto acid dehydrogenase complex and the alpha-ketoglutarate dehydrogenase complex. Contrary to our predictions, DLAT silencing was linked to an increase in lipid accumulation in adipocytes instead of the reduction we predict but this could be due to accelerated adipogenesis, which would be an interesting effect to study [63]. None of the other potential targets (Glycosylphosphatidylinositol Anchored High Density Lipoprotein Binding Protein 1 (*GPIHBP1*), *LIPA* and Alcohol Dehydrogenase 5 (*ADH5*)) were essential in any of the tested cancer cell lines, but some of them were associated to various human diseases or have homozygous null mouse models with various phenotypes [71–73]. Even if some of the 27 potential targets are associated with diseases and severe phenotypes in mice or are essential in some cancer cell lines, they should not be immediately dismissed as potential targets since the tested cell lines are not adipocytes. Furthermore, inhibition of these gene products in developed adipocytes has not been tested. Thus, at this early hypothesis generation step, any of the gene products identified have the potential to induce change in the hypertrophic adipocyte phenotype.

Söhle et al. identified genes involved in adipogenesis and fat storage in humans using siRNA targeting 7784 different genes [63]. Out of those, 333 silencing experiments had the effect of either increasing or decreasing lipid accumulation and adipogenesis. Unfortunately, the authors did not share their data. Out of the 36 genes that the authors reported as having the largest changes in expression during adipogenesis, 7 are part of the iTC1390adip metabolic network. Two of these genes (HSD17B12 and DLAT) are predicted in our work as potential targets (i.e., affecting lipid droplet formation but not biomass). As discussed above, Söhle et al. show that a HSD17B12 knock down is linked to reduced lipid accumulation in adipocytes in agreement with the predicted effect in iTC1390adip. In the case of DLAT, silencing was linked to an increase in lipid accumulation in adipocytes instead of the reduction we predict but potentially as a result of a different mechanism. More experimental work is needed to resolve the discrepant results between studies.

Multiple techniques exist to use expression data in conjunction with metabolic networks [74–76]. To our knowledge, it is the first time FBA and expression fold differences have been used in combination to restrict maximal fluxes for the various reactions of the network. Our method is limited by the fact that we only use relative differences in gene expression between the two tissues and do not take into consideration the expression levels or each enzyme’s kinetics to modulate the maximum fluxes of the reactions. Using this technique and the unrestricted media simulation, we have identified a total of 20 genes as being interesting targets for the reduction of lipid droplet production. 16 of the genes (*GAPDH, AGK, PTDSS1, LIPA, CEPT1, PCYT2, HMGCS1, FADS1, TECR, HSD17B12, ADH5, ELOVL4, AGPS, FAR2, DGAT1, LCAT*) were already identified in the previous analysis and are discussed above while the remaining 4 (Aldo-Keto Reductase Family 1, Member A1 (*AKR1A1*), Lipin 1 (*LPIN1*), Choline Phosphotransferase 1 (*CHPT1*), Carnitine Palmitoyltransferase 2 (*CPT2*)) are additional new potential targets for reducing hypertrophy in adipocytes. *CHPT1* is neither associated to a human disease nor possesses a mouse deletion model. *CPT2* deficiency leads to a broad range of phenotypes, from neonatal death to late onset of manifestations in adulthood. All forms of the disease are associated to reduced carnitine metabolism leading to various clinical symptoms from myoglobinuria and hypoglycemia to seizures and death [77]. *AKR1A1* was the only new gene identified in visceral adipose tissues due to the deletion having no effect on biomass compared to the subcutaneous tissue. *AKR1A1* deficiency is not associated to any disease in humans while mouse deletion models for *AKR1A1* present reduced levels of ascorbic acids and an accumulation of d-glucuronic acid and saccharate [78]. Lastly, *LPIN1* deficiency leads to the development of recurrent myoglobinuria before age 5 [79]. Deletions of *LPIN1* in mice leads to the development of neonatal fatty liver that resorbs between 14 and 18 days. These mice also develop peripheral neuropathies, reduced plasma leptin levels and insulin resistance associated with a reduction of 50–90% in white and brown fat pad mass [80, 81]. Higher levels of *LPIN1* in mouse adipose tissues leads to increased adiposity and higher insulin sensitivity [82]. The effect of *LPIN1* deficiency in human is restricted to muscles and is less severe than in mice due to the near ubiquitous expression in humans of 2 other *LPIN* genes, *LPIN2* and *LPIN3*, which encode related proteins [79]. *LPIN1* is necessary for the development of mature adipocytes via the induction of adipogenic genes such as peroxisome proliferator-activated receptor γ (PPARγ) and CAAT enhancer binding protein-α (C/EBPα) [83]. This indicates that some of the predicted targets might have some unpredicted effects on adipose tissue due to regulation or transcription effect which cannot be predicted using a metabolic network. Like the genes identified previously, most of these additional genes could also serve as potential targets for the modulation of fat storage and adipose tissue remodeling. Additionally, we found more than 50 different genes which may stimulate lipid droplet or biomass production when deleted in our metabolic network either in subcutaneous or visceral adipose tissue (data not shown). None of these deletions have been investigated thus far in vivo.

## Conclusions

In this work we develop iTC1390adip, an improved human adipocyte metabolic network with less inconsistencies and additional knowledge on protein complexes. The creation of iTC1390adip shows that extensive manual curation is still necessary in the development of metabolic networks. Methodologically, we optimize two objective functions, one for biomass production and another for lipid droplet production. We used our network to predict genes that could potentially reduce adipocyte hypertrophy (decrease lipid droplet production without affecting biomass production), a negative factor for adipose tissue metabolic dysfunction contributing to type 2 diabetes. We also identified genes differentially regulated between visceral and subcutaneous adipose tissues which may help explain some known differences between the two tissues.

## Additional files

Additional file 1: Results from every deletion experiment in the article as well as the assignment of gene essentiality for optimal growth from the 6 cell-lines of the two studies used in the validation of the network. (XLSX 54 kb)

Additional file 2: R code for the simulation and validation if iTC1390adip. (ZIP 452 kb)

Additional file 3: Figure_S1.  Graph of the effect of each gene’s deletion on both biomass and lipid droplet production compared to the wild type network when glucose and TAG uptake are restricted. **Table_S1.docx.** Biomass and lipid droplet constituent with stoichiometry as well as growth media definition. **Table_S2.xlsx.** List of metabolic tasks used to insure proper network behavior under various circumstances. **Table_S3.xlsx.** List of fluxes for imports and exports in the network at each time point when optimising for lipid droplet production with restrictions to the values of TAG extraction, glucose uptake and NEFA release to the experimental values from obese and lean subjects. **Table_S4.xlsx.** List of fluxes for imports and exports in the network at each time point when optimising for acetyl-CoA production with restrictions to the values of TAG extraction, glucose uptake and NEFA release to the experimental values from obese and lean subjects. **Table_S5.xlsx.** Effect of gene deletion in mouse models for the genes predicted to have an effect on adipocyte hypertrophy. **Table_S6.docx.** Number of genes having an increased effect on lipid droplet and biomass production in either of the adipose tissues compared to the other. **Table_S7.xlsx.** List of genes identified as potential targets when restricting the flux of reactions using gene fold differences between subcutaneous and visceral adipose tissues. iTC1390adip.xml and iTC1390adipRaven.xml files containing the iTC1390adip network in SDML and raven formats as described above. (ZIP 911 kb)
