# Supplementary material for: Remodeling adipose tissue through in silico modulation of fat storage for the prevention of type 2 diabetes
Source: BMC Syst Biol. 2017 Jun 12;11:60. doi: 10.1186/s12918-017-0438-9 (PMC5468946; doi:10.1186/s12918-017-0438-9)
Supplement: Supplementary file 2 — R code for the simulation and validation if iTC1390adip. (ZIP 452 kb) [file 12918_2017_438_MOESM2_ESM.zip › iTC1390adip_Rsession/READ-ME.docx]

Read-me

Here are the various step to doing the validations and tests related to the iTC1390adip network

Required packages and programs:

The sybil package is required to run the various scripts included here. A solver and it’s API package also needs to be installed, we personally used the IBM ILOG CPLEX optimization studio solver and cplexAPI R package.

Loading the network:

The iTC1390adip.Rdata contains three versions of the network used during the various validations and tests. iTC1390adipNoEx has all the exchange reactions set to 0 and is used in the various tests. iTC1390adipLD simulated the IMDM growth medium and lipid droplet formation as the objective function and iTC1390adipBIO which also simulates the IMDM growth medium but has biomass production as the objective function.

Validations:

Metabolic tests:

The Validations.R script runs all 245 metabolic tests that were used in the validation of the network. It uses the network with no active exchange and returns a list for the results of each test (1 for success and 3 for failure).

Time dependant analysis:

The Lean.R and Obese.R scripts use the network with no active exchange to integrate the data from McQuaid *et al.* Adn run simulation on the optimization of lipid droplet production and on acetyl-CoA production. The objective can be switched from lipid droplet production to acetyl-CoA production by commenting or decommenting lines 8 to 13 of each script. Each script return the objective value and the entire simulation results for each time point.

Gene Deletion Analysis:

The gene deletion analysis was eralized using the geneDeleiton function from the sybil package.
