# Supplementary material for: Remodeling adipose tissue through in silico modulation of fat storage for the prevention of type 2 diabetes
Source: BMC Syst Biol. 2017 Jun 12;11:60. doi: 10.1186/s12918-017-0438-9 (PMC5468946; doi:10.1186/s12918-017-0438-9)
Supplement: Supplementary file 3 — Graph of the effect of each gene’s deletion on both biomass and lipid droplet production compared to the wild type network when glucose and TAG uptake are restricted. Table_S1.docx. Biomass and lipid droplet constituent with stoichiometry as well as growth media definition. Table_S2.xlsx. List of metabolic tasks used to insure proper network behavior under various circumstances. Table_S3.xlsx. List of fluxes for imports and exports in the network at each time point when optimising for lipid droplet production with restrictions to the values of TAG extraction, glucose uptake and NEFA release to the experimental values from obese and lean subjects. Table_S4.xlsx. List of fluxes for imports and exports in the network at each time point when optimising for acetyl-CoA production with restrictions to the values of TAG extraction, glucose uptake and NEFA release to the experimental values from obese and lean subjects. Table_S5.xlsx. Effect of gene deletion in mouse models for the genes predicted to have an effect on adipocyte hypertrophy. Table_S6.docx. Number of genes having an increased effect on lipid droplet and biomass production in either of the adipose tissues compared to the other. Table_S7.xlsx. List of genes identified as potential targets when restricting the flux of reactions using gene fold differences between subcutaneous and visceral adipose tissues. iTC1390adip.xml and iTC1390adipRaven.xml files containing the iTC1390adip network in SDML and raven formats as described above. (ZIP 911 kb) [file 12918_2017_438_MOESM3_ESM.zip › Table_S1.docx]

**Table S1:** **Biomass and lipid droplet constituent with stoichiometry as well as growth media definition**

| Growth media^a^ | Cysteine, Isoleucine, Leucine, Tryptophan, Methionine, Valine, Phenylalanine, Threonine, Histidine, Lysine. Riboflavin, Choline, Pyridoxine, Pantothenate, Thiamine, Chloride, Folate, Ca2+, Fe2+, Nitrite, Na+, K+, HCO3-, Glucose, Inositol, O2, Linoleate, Linolenate, Triacylglycerol (Chylomicron and VLDL) and Cholesterol |
| --- | --- |
| Biomass^b^ | 20.650823 H_2_O + 20.704451 ATP + 0.385872 Glutamate + 0.352607 Aspartate + 0.279425 Asparagine + 0.505626 Alanine + 0.046571 Cysteine + 0.325996 Glutamine + 0.538891 Glycine + 0.392525 Serine + 0.31269 Threonine + 0.592114 Lysine + 0.35926 Arginine + 0.153018 Methionine + 0.126406 Histidine + 0.159671 Tyrosine + 0.286078 Isoleucine + 0.545544 Leucine + 0.013306 Tryptophan + 0.259466 Phenylalanine + 0.412484 Proline + 0.352607 Valine + 0.036117 GTP + 0.039036 CTP + 0.053446 UTP + 0.009898 dGTP + 0.009442 dCTP + 0.013183 dATP + 0.013091 dTTP + 0.275194 glucose-6-phosphate + 0.020401 cholesterol + 0.005829 Phosphatidylserine pool + 0.017486 Sphingomyelin pool + 0.002914 Phosphatidylglycine pool + 0.011658 Cardiolipin pool + 0.154463 Phosphatidylcholine pool + 0.055374 Phosphatidylethanolamine pool + 0.023315 Phosphatidylinositol pool |
| Lipid Droplet^c^ | 0.19 1,2-diacylglycerol pool + 0.0014 1-acyl-PE pool + 0.0024 1-radyl-2-acyl-sn-glycero-3-phosphocholine + 0.0006 2-lysolecithin pool + 0.005 cholesterol + 0.34 cholesterol-ester pool + 0.005 fatty acid pool + 0.0008 O-1-alk-1-enyl-2-acyl-sn-glycero-3-phosphoethanolamine + 0.0092 Phosphatidylcholine pool + 0.0034 Phosphatidylethanolamine pool + 0.0016 phosphatidylinositol pool + 0.0002 Phosphatidylserine pool + 0.0004 Sphingomyelin pool + 0.44 Triacylglycerol pool |

The biomass reaction also produces 20.650823 units of ADP, H+ and inorganic phosphate.

^a^ No stoichiometry is given for the elements in the growth medium, they are considered as having an infinite availability during simulations.

^b^ The biomass reaction is derived from the biomass reaction of the Recon2 metabolic network.

^c^ The lipid droplet composition is kept from the iAdipocytes1809 metabolic network.
