# Supplementary figures and images for: Remodeling adipose tissue through in silico modulation of fat storage for the prevention of type 2 diabetes
Source: BMC Syst Biol. 2017 Jun 12;11:60. doi: 10.1186/s12918-017-0438-9 (PMC5468946; doi:10.1186/s12918-017-0438-9)

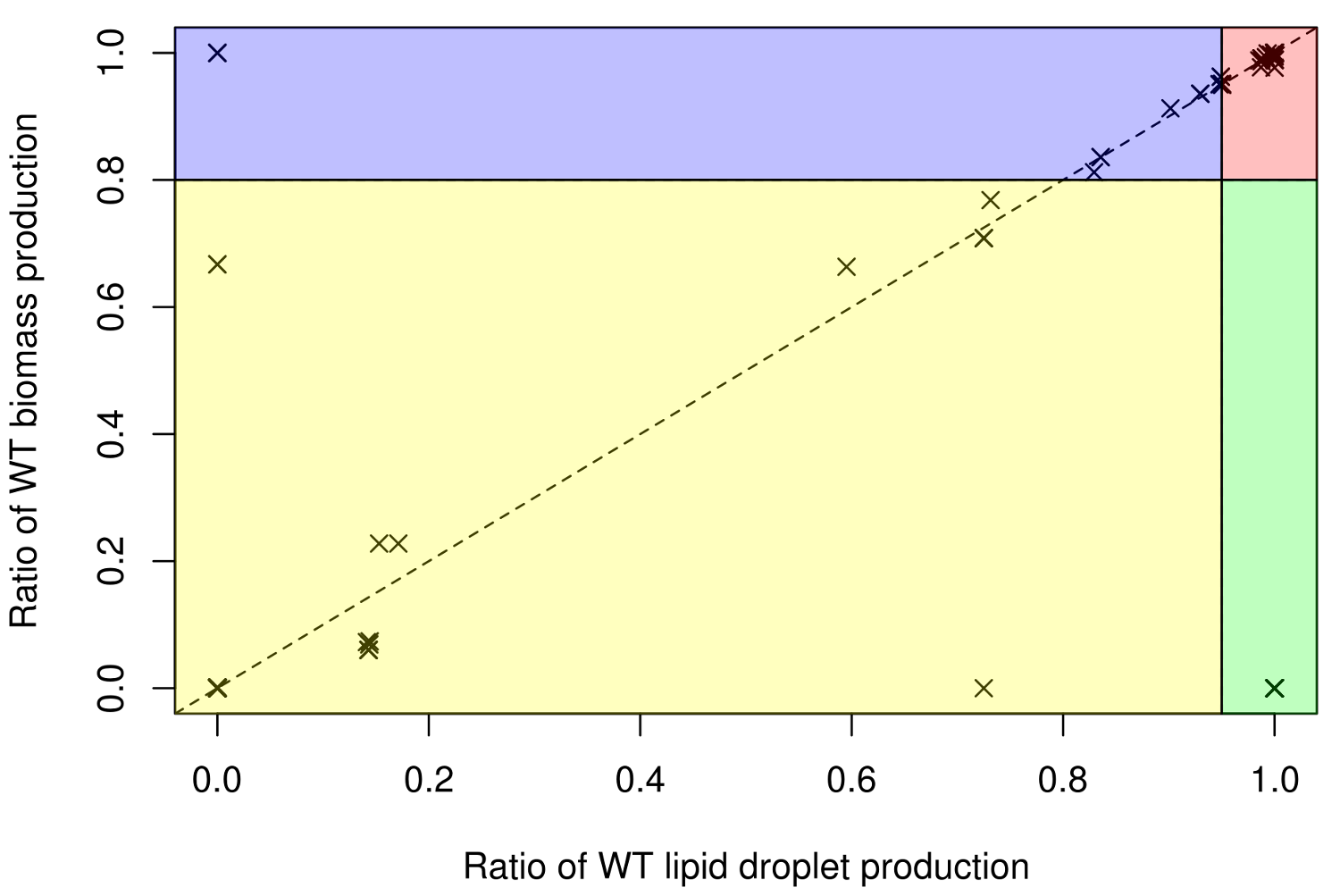

Supplement: Supplementary file 3 — Graph of the effect of each gene’s deletion on both biomass and lipid droplet production compared to the wild type network when glucose and TAG uptake are restricted. Table_S1.docx. Biomass and lipid droplet constituent with stoichiometry as well as growth media definition. Table_S2.xlsx. List of metabolic tasks used to insure proper network behavior under various circumstances. Table_S3.xlsx. List of fluxes for imports and exports in the network at each time point when optimising for lipid droplet production with restrictions to the values of TAG extraction, glucose uptake and NEFA release to the experimental values from obese and lean subjects. Table_S4.xlsx. List of fluxes for imports and exports in the network at each time point when optimising for acetyl-CoA production with restrictions to the values of TAG extraction, glucose uptake and NEFA release to the experimental values from obese and lean subjects. Table_S5.xlsx. Effect of gene deletion in mouse models for the genes predicted to have an effect on adipocyte hypertrophy. Table_S6.docx. Number of genes having an increased effect on lipid droplet and biomass production in either of the adipose tissues compared to the other. Table_S7.xlsx. List of genes identified as potential targets when restricting the flux of reactions using gene fold differences between subcutaneous and visceral adipose tissues. iTC1390adip.xml and iTC1390adipRaven.xml files containing the iTC1390adip network in SDML and raven formats as described above. (ZIP 911 kb) [file 12918_2017_438_MOESM3_ESM.zip › Figure_S1.tiff]
